# Medical Applications and Advancement of Near Infrared Photosensitive Indocyanine Green Molecules

**DOI:** 10.3390/molecules28166085

**Published:** 2023-08-16

**Authors:** Zulpya Mahmut, Chunmei Zhang, Fei Ruan, Nan Shi, Xinyao Zhang, Yuda Wang, Xianhong Zheng, Zixin Tang, Biao Dong, Donghui Gao, Jiao Sun

**Affiliations:** 1 Department of Cell Biology and Medical Genetics, College of Basic Medical Science, Jilin University, Changchun 130021, China; zlpym22@mails.jlu.edu.cn (Z.M.); zhangcm499@jlu.edu.cn (C.Z.); xinyaoz22@mails.jlu.edu.cn (X.Z.); wyd21@mails.jlu.edu.cn (Y.W.); zhengxh@jlu.edu.cn (X.Z.); 2 State Key Laboratory on Integrated Optoelectronics, College of Electronic Science and Engineering, Jilin University, Changchun 130012, China; ruanfei1919@mails.jlu.edu.cn (F.R.); tangzx1920@mails.jlu.edu.cn (Z.T.); 3 Department of Respiratory Medicine, No. 964 Hospital of People’s Liberation Army, 4799 Xi’an Road, Changchun 130062, China; 13504327085@163.com; 4 Department of Anesthesiology and Operating Room, School and Hospital of Stomatology, Jilin University, Changchun 130012, China

**Keywords:** ICG, composite nanoparticles, light therapy, tumor treatment, antibacterial treatment

## Abstract

Indocyanine green (ICG) is an important kind of near infrared (NIR) photosensitive molecules for PTT/PDT therapy as well as imaging. When exposed to NIR light, ICG can produce reactive oxygen species (ROS), which can kill cancer cells and pathogenic bacteria. Moreover, the absorbed light can also be converted into heat by ICG molecules to eliminate cancer cells. In addition, it performs exceptionally well in optical imaging-guided tumor therapy and antimicrobial therapy due to its deeper tissue penetration and low photobleaching properties in the near-infrared region compared to other dyes. In order to solve the problems of water and optical stability and multi-function problem of ICG molecules, composite nanomaterials based on ICG have been designed and widely used, especially in the fields of tumors and sterilization. So far, ICG molecules and their composite materials have become one of the most famous infrared sensitive materials. However, there have been no corresponding review articles focused on ICG molecules. In this review, the molecular structure and properties of ICG, composite material design, and near-infrared light- triggered anti-tumor, and antibacterial, and clinical applications are reviewed in detail, which of great significance for related research.

## 1. Introduction

Traditional treatments for tumors typically involve surgery, radiation, and chemotherapy. However, these approaches often result in high recurrence and mortality rates due to factors such as distant metastases, drug resistance, and inevitable side effects on normal tissues. Similarly, bacterial infectious diseases, including periodontal disease, septic liver abscess, keratitis [1], and diabetic foot ulcers [2], present significant challenges. Traditional treatments such as mechanical debridement combined with antibiotic therapy are commonly used for periodontal disease. However, due to antibiotic resistance and the presence of deep lesions that cannot be effectively eradicated by conventional treatments, alternative approaches are needed. Extensive preclinical and clinical research is being conducted on phototherapy, resulting in a light-activated local therapeutic technique that encompassing photodynamic therapy (PDT) and photothermal therapy (PTT). Near-infrared (NIR) light with a wavelength of 700–1100 nm is considered the optimal light source for both antimicrobial photodynamic therapy (aPDT) and PTT due to its excellent tissue penetration and minimal damage to healthy tissues. To address these challenges, the application of NIR-responsive photosensitizers and photothermal agents shows great promise.

**Figure 1:**
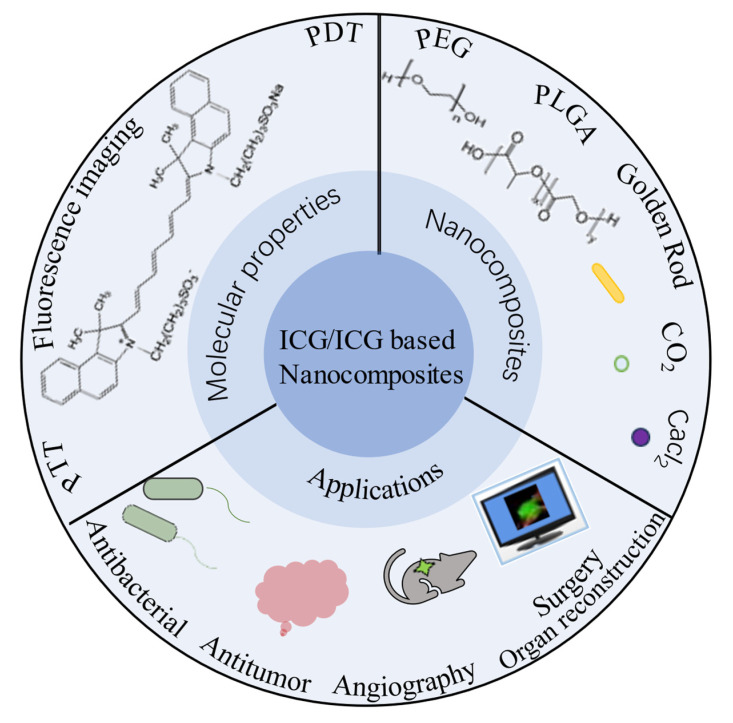
ICG Nanocomposite and its Applications.

ICG is an anthocyanine compound that exhibits strong light absorption in the NIR region and possesses fluorescent properties. Its primary application is oncology diagnosis and treatment. Intraoperative ICG imaging can assist in identifying different types of solid tumors and metastases [3]. Furthermore, ICG has the potential to induce the death of cancerous tissues through the generation of reactive oxygen species (ROS) and/or by exerting thermotherapeutic effects when exposed to radiation. However, ICG is subject to drawbacks such as water instability, photobleaching properties, photodegradability, thermal degradation [4,5,6], very short circulatory lifetime, and a tendency to bind to lipoproteins, resulting in rapid clearance in vivo [7], which restrict its clinical application. Consequently, several formulation strategies have been explored to overcome these challenges. For instance, inorganic and polymeric nanoparticles (NPs) and liposomes are involved in encapsulating ICG [8], and hybrid cell membranes have also been employed to enhance therapeutic efficacy. Therefore, it is important to create a drug delivery system that contains ICG in order to enhance the in vivo stability, tumor targeting and therapeutic effectiveness. Over the years, many methods have been applied to design ICG composite structures to enhance their biocompatibility and enhance their versatility. Moreover, ICG has unique PDT, PTT, and imaging functions, all of which are generated through NIR light excitation. Therefore, how to utilize energy and optimize the corresponding function are also the focus of research. In terms of clinical application, the integration of nanoparticle-based platforms in treatment also holds new promise for patients. There have been many related studies, but there are still no review articles on ICG molecules, which is lacking in this area. Figure 1 illustrates the concept of designing ICG and its composite NPs for accurate and personalized medicine.

## 2. Molecular Structure 

**Figure 2:**
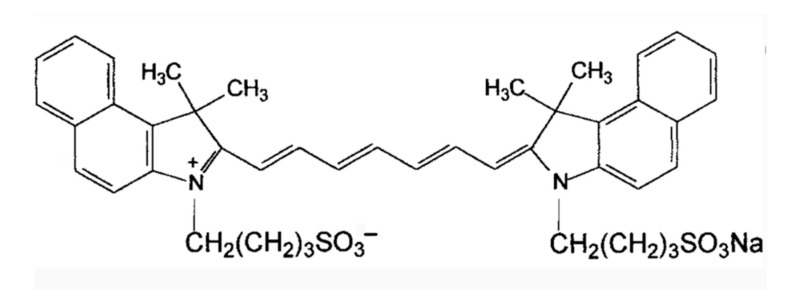
Chemical structure of ICG [4].

ICG is an amphiphilic molecule, meaning it has both hydrophilic and lipophilic properties [4,9]. It is composed of two polycyclic parts (benzindotricycin) that are predominantly lipophilic and connected by carbon chains as depicted in Figure 2. Each polycyclic part is bound to a sulfate group, which provides some water solubility to ICG. This property allows ICG to bind to both hydrophilic and lipophilic substances, including phospholipids, thereby enhancing its fluorescence intensity. The fluorescence yield of ICG is influenced by these binding interactions.

At concentrations below 5 µM, ICG exists as a monomer. However, at concentrations exceeding 100 µM, aggregation occurs, the absorption peak of the monomeric form of ICG is around 785 nm, whereas the maximum absorbance of the aggregates shifts to approximately 690 nm at concentrations greater than 100 µM [9]. ICG J-aggregates (IJA), a derivative of ICG, exhibits a dark green color visible to the naked eye, while ICG appears light- green. IJA also undergoes a significant red shift of approximately 100 nm compared to ICG. In various media, both IJA and ICG display a characteristic peak at 892 nm. IJA demonstrates greater water stability and does not undergo significant spectral changes induced by salt [10], unlike ICG. Under physiological conditions, ICG molecules undergo aggregation and polymerization with plasma proteins or lipoproteins after intravenous injection, this results in the main peak of the absorption spectrum red-shifting to around 805 nm or 810 nm, leading to a relatively stable spectrum [9]. This suggests that ICG molecules have different optical and stability characteristics when they are in water or coupled to proteins and lipids in vivo. It also suggests that the fluorescence intensity varies depending on the concentration.

## 3. Design of ICG and Composite Nanomaterials

ICG molecules offer significant advantages in terms of biosafety and metabolic modalities. ICG has been clinically approved and is considered safe for use. However, its practical application is limited due to drawbacks such as photobleaching and short blood circulation half-life (t_1/2_ = 2~4 min). Moreover, ICG NIR fluorescent dyes are water-soluble compounds that exhibit chemical instability in aqueous media. They tend to aggregate at high concentrations, bind to proteins under physiological conditions, and undergo photodegradation, thermal degradation, or photobleaching. Therefore, achieving stability in aqueous media is a prerequisite for their biomedical application to maximize their benefits. To address these challenges, efforts have been made to incorporate or polymerize ICG NIR fluorescent dyes into nanoparticles. Compared to free ICG molecules, ICG encapsulated in nanostructures offer several advantages:

1. Enhanced stability in physiological environments;
2. Substantially higher photothermal conversion efficiency;
3. Prolonged circulation duration in the bloodstream due to the development of nanostructures. Additionally, the enhanced permeability and retention (EPR) effect of solid tumors can potentially facilitate tumor targeting;
4. The nanoparticle platform allows for the combination of various diagnostic and therapeutic tools. For instance, simultaneous loading of chemotherapeutic drugs and NIR dyes can be achieved, enabling the concurrent use of photothermal therapy and photothermal-regulated drug therapy to enhance tumor treatment.

This approach of synthesizing ICG within a nanocomposite framework presents a promising avenue for improving and harnessing the potential of ICG in photothermal and photodynamic therapy.

### 3.1. ICG/MOFs Nanoparticles

Metal-organic frameworks (MOFs) are hybrid porous polymers constructed from metal ions/clusters and multi-layered organic ligands. They possess unique properties such as a large surface area, tunable pore size and shape, tunable composition, and functionalized pore surfaces, which make them highly advantageous over conventional porous materials. These properties have led to their utility in applications such as drug adsorption, delivery, and release [11,12].

**Figure 3:**
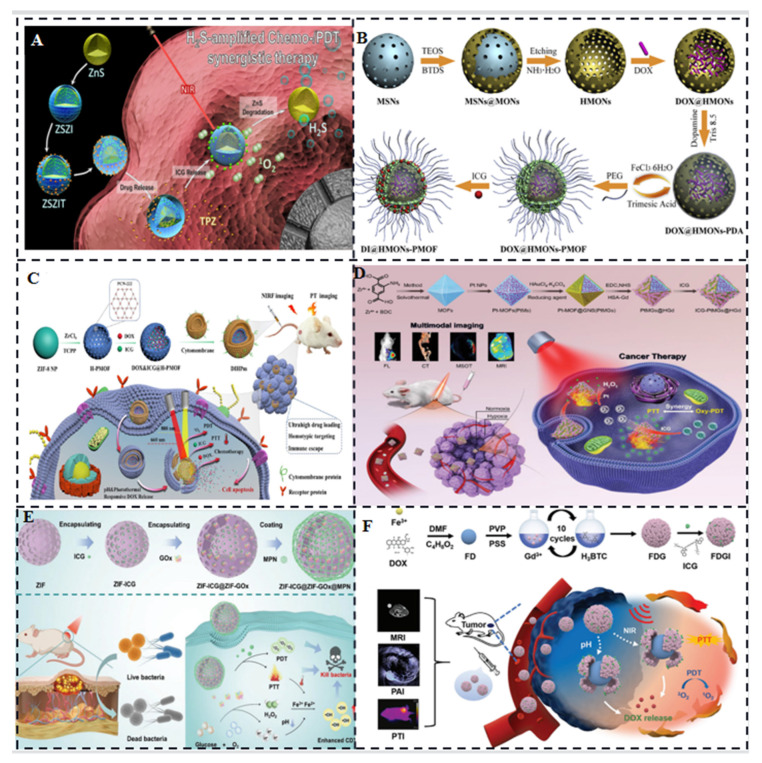
(**A**) Schematic of ZSZIT nanosystem with high loading ICG for combined H_2_S amplified phototherapy/chemotherapy [14]. (**B**) Nanoplatform with big cavity and porous network structure for optimal phototherapy/chemotherapy synergy and high drug loading [15]. (**C**) Schematic illustrating the synergistic use of the cancer cell membrane-encapsulated bionic agent DIHPm16 and DOX/ICG co-delivery [16]. (**D**) Nanoplatform (ICG-PtMGs@HGd) that targets tumor hypoxia limitation in order to improve ICG photodynamics and cause cell death [18]. (**E**) Nanoplatform carrying GOX/Metal Polyphenol Network (MPN) for continuous oxygen generation and alteration of bacterial infection site PH to enhance synergistic phototherapeutic/chemodynamic effects involving ICG [19]. (**F**) PDT/PTT combination therapy with Fe-DOX@Gd-MOF-ICG [20].

One example of MOF application is ZIF-8 addressing challenges related to photosensitizers. ZIF-8 relieves hypoxia, slows the self-aggregation of photosensitizers, and permits photodynamic ROS production to permeate deeper into the tissue [13]. Fang C et al. designed ZIF-8-coated ZnS nanoparticles for the co-delivery ICG and tirapazamine (TPZ) for H_2_S amplified cooperative therapy (Figure 3A) [14]. ZIF-8 is capable of obtaining high ICG loading, decomposing, and releasing ICG in acidic environments due to its ultra-high porosity, adjustable structure, and biodegradable qualities under acidic circumstances.

Another study by Chen et al. combined MOFs with polydopamine (PDA) intercalated hollow mesoporous organosilica nanoparticles (HMONs) to form molecular organic/inorganic hybrid nanocomposites (HMONs-PMOF). They loaded doxorubicin hydrochloride (DOX) and ICG into the inner cavity of HMONs and the outer porous shell of MOF, respectively, creating DI@HMONs-PMOF (Figure 3B) [15]. This approach achieved high drug loading efficacy, effective ICG photothermal effect, and pH/NIR-triggered DOX release for chemotherapeutic synergy.

Hollow nanostructures have also attracted interest in drug delivery systems due to their high drug-carrying capacity and unique physicochemical properties. Sun et al. formed a shell of Zr-based porphyrin MOF through the digestion of prefabricated ZIF-8 nanoparticle templates and coordination-driven self-assembly of tetra (4-carboxyphenyl) porphyrin (TCPP) ligands and Zr^4+^ ions. They encapsulated DOX and ICG into the resulting hollow spherical nanoparticles with mesoporous MOF shells (DIHP) (Figure 3C) and further encapsulated them with mouse breast cancer (4T1) cell membranes to achieve imaging-guided synergistic cancer therapy [16].

In another study, ZIF-8 nanoparticles were equipped with ICG and cytochrome c (Cyt c) by Jiang et al. Cyt c, possessing peroxidase-like activity, induces programmed cell death and decomposes H_2_O_2_ to O_2_, enhancing the PDT efficiency of ICG molecules [17]. To address the limitations posed by the oxygen-depleted tumor microenvironment, You et al. constructed an on-demand O_2_ self-oxygenating nanoplatform, MOF@GNSs (PtMGs). Octahedral gold nanoshells (GNSs) with platinum (Pt) nanase-modified MOFs served as internal templates, chelated with human serum albumin functionalized with gadolinium (HSA-Gd) and loaded with ICG (ICG-PtMGs@HGd) (Figure 3D). This platform enabled synergistic PDT/PTT effects and fluorescence/multispectral photoacoustic tomography/X-ray computed tomography/magnetic resonance imaging [18]. Moreover, Fu et al. doped ICG and glucose oxidase (GOx) into homologous ZIF-8 nanoparticles coated with a metallopolyphenol network (MPN) consisting of Fe^3+^ and tannic acid (TA) (Figure 3E). This method lowers pH and continuously replenishes H_2_O_2_ in order to simultaneously implement PTT, PDT and chemodynamic therapy (CDT) for ICG, which improves bactericidal efficiency and expands the antimicrobial spectrum [19]. With a self-assembled synthetic MOF readsorption made of Gd^3+^ ions and 1,3,5-benzenetricarboxylic acid (H_3_BTC) wrapped in Fe^3+^ ions and doxorubicin hydrochloride (DOX) molecules (Figure 3F), Zhu et al. created the photoacoustic and photothermal imaging platform Fe-DOX@Gd-MOF-ICG [20]. After 10 cycles of reactions, average hydrodynamic nanoparticle diameter rose from 150 nm (for FD NPs) to 250 nm (for FDG NPs), according to Dynamic Light Scattering (DLS) measurements. Although many multifunctional MOFs capable of carrying and releasing various compounds have been designed, MOF-based nanoparticles differ in size and surface chemistry and their intracellular transport pathways.

### 3.2. ICG/Polymers

The encapsulation of ICG molecules within various biodegradable polymers offers several advantages for ICG, including improved stability, prevention of aggregation, and reduced fluorescence quenching. Polymeric carriers provide benefits during ICG delivery, such as enhanced deep tissue permeability, generation of singlet oxygen upon light exposure, and minimal autofluorescence. Additionally, polymers can extend the half-life of ICG, reduce its rapid degradation, and improve its biocompatibility, biodegradability, fluorescence intensity, physicochemical stability, target specificity, and pharmacokinetic properties.

**Figure 4:**
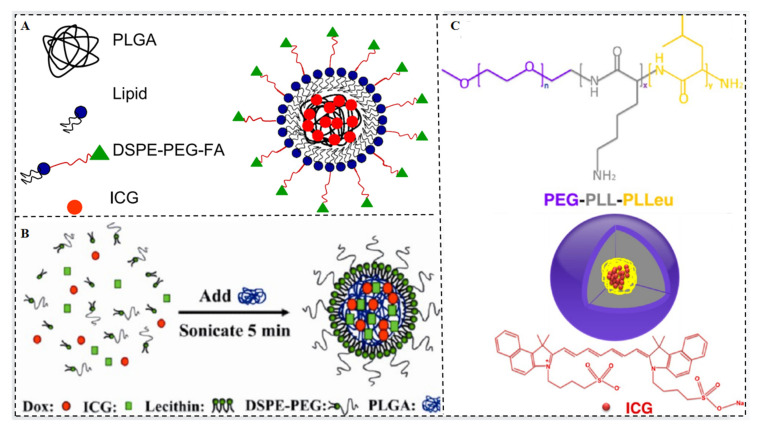
(**A**,**B**) Connecting ICG’s micelles [24,26]. (**C**) Assembly of ICG and PEG-PLL-PLLeu [28].

Different polymers have been utilized to encapsulate and deliver ICG, including poly (D,L-lactic-co-glycolic acid) (PLGA), polyethylene glycol (PEG), and poly (ε-caprolactone) (PCL). These polymers have been employed in various applications such as imaging, diagnostics, and therapeutics. However, each polymer has its advantages and limitations [21]. Lipid-polymer hybrid nanoparticles (LPNs) are core-shell structures composed of a polymer core and a lipid or lipid-PEG shell. LPNs combine the advantages of both polymer nanoparticles and liposomes [22], particularly in terms of physical stability and biocompatibility [23]. Recent studies have shown that LPNs derived from polymeric nanoparticles and liposomes exhibit superior cellular delivery efficacy in vivo compared to LPNs derived solely from polymers or liposomes. For example, folate receptor-targeted, ICG dye-doped PLGA lipid NPs (FA-ICG-PLGA-lipid NPs) demonstrated excellent anti-photobleaching stability, specific tumor targeting, and extended circulation times (Figure 4A) [24].

To enhance the antitumor efficacy of ICG, it is crucial to improve its accumulation in tumors by controlling the nanoparticle size. Zhao et al. developed ICG-PLGA-lecithin-PEG core-shell nanoparticles (INPs) with sizes of 39 nm, 68 nm, and 116 nm using a one-step nanoprecipitation method [25]. INPs at 39 nm demonstrated increased absorption into pancreatic cancer tumor cells and improved phototherapeutic effects in vitro when compared to bigger diameters (68 nm and 116 nm).

DOX and ICG-loaded PLGA-lecithin-PEG nanoparticles (DINPs) were prepared by Zheng et al. using a single-step ultrasound method. Synergistic treatment with DINPs demonstrated improved apoptosis and cell death compared to chemotherapy or photothermal treatment alone (Figure 4B) [26]. In addition to polymeric nanoparticles and lipid-polymers, polymeric nanocapsules have been designed to encapsulate ICG. These nanocapsules consist of polyallylamine hydrochloride (PAH) chains and are covalently coated with PEG via a reductive amination reaction [27]. The encapsulation of ICG in these constructs prolongs its circulation time and retards hepatic accumulation, addressing the rapid elimination of ICG from the vascular system. Furthermore, polymeric micelles composed of amphiphilic PEG-peptide hybrid triblock copolymers, such as polyethylene glycol-b-poly (l-lysine)-b-poly (l-leucine) (PEG-PLL-PLLeu), have been utilized for ICG delivery. Electrostatic attraction holds the hydrophobic portion of the ICG to the hydrophobic micelle core and the hydrophilic head (Figure 4C) [28]. ICG is more readily taken up and maintained by cells when bound to micelles, and its quantum yield is relatively high. These studies highlight the potential of hybrid polymers, liposomes, and micelles as effective carriers for ICG molecules, providing enhanced stability, controlled release, improved biodistribution, and therapeutic efficacy.

### 3.3. Liposome-Coated ICG

Liposome-encapsulated ICG has been developed to address the limitations of ICG, such as lack of targeting, easy quenching, self-aggregation, and instability. Liposomes are lipid bilayer structures that can encapsulate hydrophobic compounds like ICG, providing stability and controlled release. Here are some key findings from studies using liposome-encapsulated ICG.

**Figure 5:**
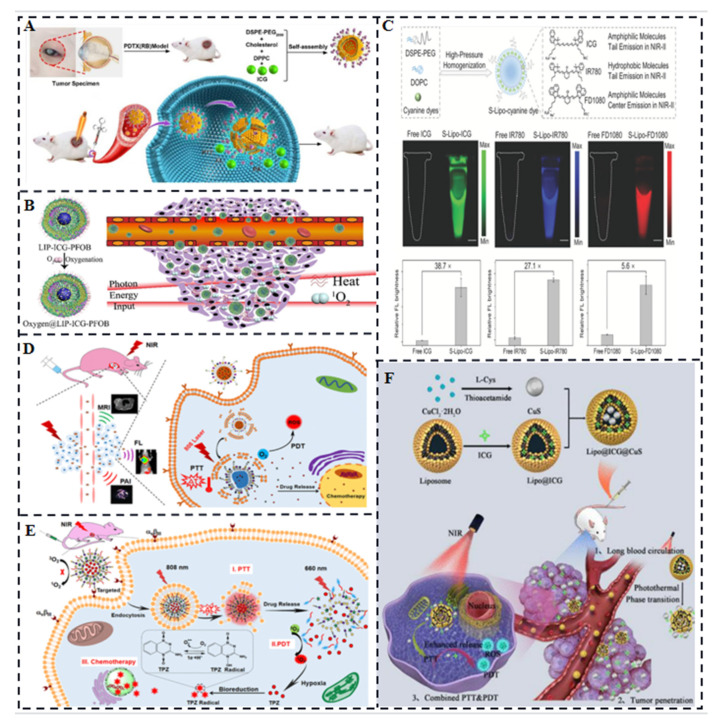
(**A**) Ocular detection and treatment using the photothermal/fluorescent/photoacoustic imaging capabilities of the FDA-approved contrast agent ICG [29]. (**B**) Oxygen-carrying capacity of perfluorooctane bromide enhances aPDT treatment with PA/FL imaging lead-in [30]. (**C**) Liposome-encapsulated ultra-bright fluorophores display 38.7 times brighter NIR-II than free ICG and last up to 30 min for cerebrovascular imaging [32]. (**D**) ICG, DOX, and conjugated gadolinium chelates are released synergistically as a result of photothermal stimulation of thermosensitive liposomal phase transition [33]. (**E**) ICG transmits energy to activate Ce6, which then consumes oxygen and creates a hypoxic state that triggers the anticancer medication TPZ action [34]. (**F**) Novel nanoparticles consisting of thermosensitive liposomes, ICG and CuS NPs [35].

Imaging-guided PTT of retinoblastoma (Rb). Liu et al. synthesized ICG encapsulated in lipid bilayer multifunctional liposomes (ILP) for the treatment of Rb (Figure 5A) [29]. ILP exhibited better tumor targeting and higher temperature elevation compared to free ICG when irradiated with near-infrared laser. ILP showed improved imaging and PTT outcomes for Rb. Sheng et al. combined a biocompatible chemical, perfluorooctyl bromide (PFOB), with ICG in a nanoliposome structure via a simple two-step emulsion method [30]. This ICG & PFOB co-loaded nanoliposomes (LIP-PFOB-ICG) enabled in vivo computed tomography (CT) contrast imaging, which provided better anatomical information of the tumor compared to PA and FL imaging with ICG. More importantly, due to the excellent oxygen-carrying capacity of PFOB, it effectively alleviates tumor hypoxia and improves the collisional energy transfer efficiency between ICG and oxygen. Li et al. also constructed a hollow gold nanosphere (FAL-ICG-HAuNS) affixed with pardaxin (FAL) peptide targeting the ER, modified indocyanine green (ICG), and oxygen-transferring haemoglobin (Hb) liposomes (FAL-Hb lipids) comprising a nanoplatform for reversing the hypoxic microenvironment [31], loaded with ICG also overcomes the critical factor of limited therapeutic efficacy due to the existence of a competitive relationship between imaging and phototherapy. Gao et al. developed liposome-encapsulated cyanine dyes for NIR-II imaging. Improved cerebrovascular imaging was made possible by liposome-encapsulated ICG, which demonstrated a much higher NIR-II brightness than free ICG. Liposome-encapsulated ICG also allowed successful imaging-guided tumor surgery in a rabbit model (Figure 5C) [32]. For combination therapy and tri-modal imaging, in thermosensitive liposomes, Dai et al. administered ICG together with the anticancer medication DOX. The liposomes were modified with folic acid and conjugated gadolinium chelate for active targeting and magnetic resonance imaging (Figure 5D). This liposomal system enabled combination therapy (chemotherapy, PTT, and PDT) and tri-modal imaging guidance [33].

For synergistic PTT and PDT, in order to have combined anticancer effects, Dai et al. liposomally encapsulated ICG and the hypoxia-activated prodrug tirapamycin (TPZ). Photothermal effect of ICG, combined with photodynamic effect of released Ce6, resulted in cytotoxic singlet oxygen generation. The hypoxia caused by this process activated the antitumor activity of TPZ (Figure 5E) [34]. For photo-triggered decomposable delivery system, Gao et al. developed a nanoparticle system (Lipo@ICG@CuS) consisting of liposomes (Figure 5F). The photothermal ability of CuS NPs caused temperature to rise in response to near-infrared laser irradiation, which led the liposomes′ structural integrity to break down and the release of ICG and CuS NPs. Synergistic PDT and PTT were made possible by this technique [35].

In conclusion, liposome-encapsulated ICG has shown promising results in addressing the limitations of free ICG, enhancing fluorescence and optical intensity, improving stability, and achieving improved targeting, accumulation, and therapeutic outcomes in various applications.

### 3.4. ICG-Based Micelles Composites

**Figure 6:**
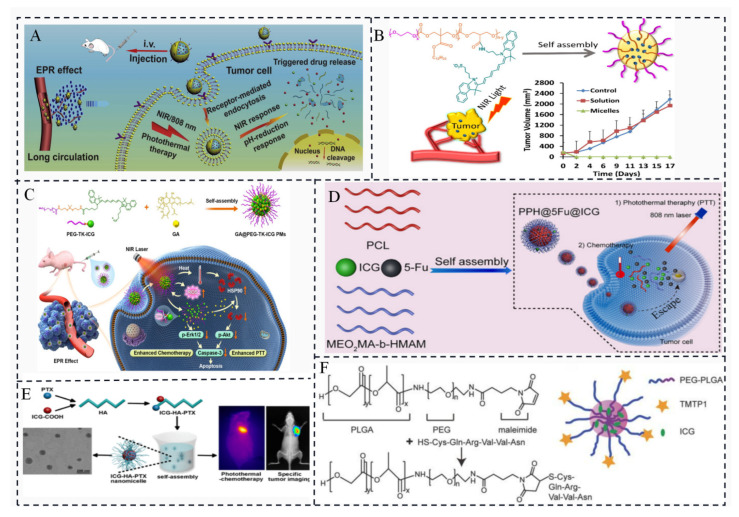
(**A**) Acid and GSH degradation release DOX and ICG from polymeric micelles [36]. (**B**) ICG covalently bound high loading micelles [37]. (**C**) ICG-bound and GA-loaded polymeric micelles for local chemotherapy–photothermal synergistic therapy of breast cancer [38]. (**D**) Mechanism of PPH@5Fu@ICG for GC [39]. (**E**) Preparation of ICG-HA-PTX and imaging-guided photothermal effect [40]. (**F**) Synthesis and structure of ITM [41].

Multifunctional pH/reduction dual-response drug delivery system: Zhang et al. developed a drug delivery system based on polymer micelles that responded to both pH and reduction stimuli. The micelles encapsulated the chemotherapeutic drug DOX and the photosensitizer ICG for NIR imaging and targeted chemotherapy-photothermal combination therapy (Figure 6A) [36]. The micelles remained stable during blood circulation, accumulated in tumors through enhanced permeation and retention (EPR) effects, and released the drugs in response to acid hydrolysis and high glutathione (GSH) concentrations. ICG enhanced cellular uptake and accelerated drug release under 808 nm light irradiation. Covalent conjugation for enhanced tumor accumulation: Covalent conjugation of ICG-NH_2_ to poly (ethylene glycol) -block-poly (2-methyl-2-carboxy-propenyl carbonate) (PEG-PCC) copolymers were reported to self-assemble into highly loaded micelles of ICG (Figure 6B). This covalent action enhanced tumor accumulation of ICG in vivo, as demonstrated by NIR imaging. The micelles showed improved therapeutic efficacy when combined with NIR irradiation, leading to complete tumor regression [37]. Polymer micelles for combined chemotherapy and PTT: Yang et al. prepared polymer micelles loaded with the chemotherapeutic agent glyburic acid (GA) and coupled with ICG. Under 808 nm laser irradiation, ICG in the micelles enabled PTT and generation of reactive oxygen species, which cleaved the TK bond of the polymer, causing the micelles to break down and release GA. GA inhibited heat shock protein 90 (HSP90) (Figure 6C), enhancing the PTT efficiency of ICG. This combination of PTT and chemotherapy showed potential for complete tumor destruction [38].Zhou et al. designed nanocomposites consisting of a poly (ε-caprolactone) (PCL) core and a poly (2-methoxyethyl acrylate-block-hydroxylpropyl methacrylamide) (P(MEO2MA-b-HMAM)) shell (Figure 6D). The nanocomposites loaded the anti-gastric cancer drug 5-fluorouracil (5Fu) and the photothermal agent ICG to form self-assembled thermosensitive micelles. Under near-infrared irradiation, ICG converted light energy into heat energy, causing the micelles to change from a gel phase to a liquid crystal phase and release the chemotherapeutic drug. This design aimed to overcome chemotherapy-induced immune escape and reduce the side effects of chemotherapy [39]. ICG derivative for tumor therapy and diagnosis: Su et al. chemically modified an ICG derivative (ICG-COOH) and paclitaxel (PTX) into hyaluronic acid (HA) backbone self-assembled nanomicelles (Figure 6E). The release of the drug from the micelles was triggered by esterase overexpressed in tumor cells, leading to the restoration of ICG fluorescence imaging properties [40]. This approach aimed to achieve multifunctional tumor therapy and diagnosis based on organic near-infrared dyes. Tumor metastasis-targeting micelles: Wei et al. developed polyethylene glycol-poly (lactic-co-glycolic acid) (PEG-PLGA) micelles modified with a tumor metastasis-targeting peptide (TMTP1) and loaded with ICG (Figure 6F). The micelles specifically accumulated in tumor metastatic sentinel lymph nodes (T-SLNs) and could be distinguished from normal sentinel lymph nodes (N-SLNs) using NIR fluorescence imaging [41].

These studies demonstrate the small size, ease of assembly and versatility of polymer micelles as drug delivery platforms for ICG and highlight their potential for combined therapy and imaging applications.

### 3.5. ICG/Gold Nanocomposites

**Figure 7:**
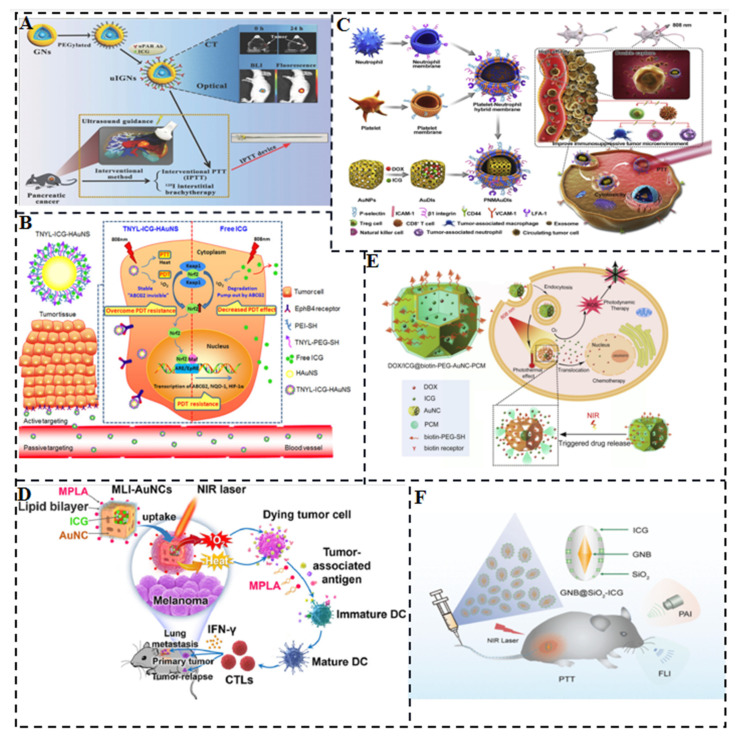
(**A**) Bioimaging-guided interventional photothermal therapy [44]. (**B**) Anti-tumor diagram of TNYL-ICG-HAuNS [45]. (**C**) PNMAuDIs treatment for breast cancer metastasis [46]. (**D**) Nanomaterials MLI-AuNCs for inhibiting recurrence of aggressive melanoma. inhibit melanoma-associated antigens, resulting in the inhibition of DC maturation and IFN-γ, and thus inhibit the recurrence of aggressive melanoma [47]. (**E**) Preparation of DOX/ICG@Biotin-PEG-AuNC-PCM for chemotherapy/photodynamic therapy [48]. (**F**) Gold-nano bipyramidal nanootheranostics for imaging and phototherapy [49].

The higher temperature mediated by light exposure significantly accelerates the photodegradation of ICG in aqueous solution through thermal decomposition. Therefore, the stability and good biocompatibility of gold (Au)-based nanomaterials such as gold nanorods (Au NRs), gold nanoparticles (Au NPs), and hollow gold nanospheres (HAuNS) have been extensively studied for enhancing the stability of ICG [42,43]. Due to the deep light penetration of ICG in the near-infrared region, it can be used for the treatment of deeply buried tumors with minimal damage to normal tissues and excellent fluorescence imaging ability. Gold nanoshells (GNs) have been approved for clinical trials to evaluate their efficacy in patients with refractory and/or recurrent head and neck tumors. Hu et al. developed an interventional photothermal therapy (IPTT) to kill pancreatic cancer (PC) cells deep in the abdominal cavity. IPTT involves inserting a NIR fiber through an 18 G percutaneous transhepatic cholangiopancreatography (PTC) puncture needle, allowing access to deep PC tissue (Figure 7A). They selected gold nanoshells coupled with anti-urokinase-type fibrinogen activator receptor (uPAR) antibody, polyethylene glycol, and indocyanine green (uIGNs) as the PTT reagents. CT and fluorescence imaging can be used for tumor visualization and to mediate PTT of PC. The first systematic comparison of the efficacy of clinical iodine-125 (125I) tissue interposition radiotherapy (IBT-125-I) and IPTT was performed in a human pancreatic cancer in situ transplantation tumor model. The results showed that the median survival rate of IPTT with one-time interventional complete ablation was improved by 25% compared to IBT-125-I [44]. Li et al. reported a nanosystem (TNYL-ICG-HAuNS) that combines NIR light-mediated PDT and PTT for specific tumor targeting. The nanosystem consisted of HAuNS modified with TNYL-PEG-SH and PEI-SH, conjugated with ICG [45]. HAuNS have been widely used for tumor-targeted delivery and drug-triggered release and have strong photothermal properties under NIR light irradiation due to their excellent surface plasmon resonance (SPR) in the NIR region. TNYL peptides interact with receptors to enhance tumor accumulation. Upregulation of nuclear factor erythroid 2-related factor 2 (NFE2L2, Nrf2) induced a significant increase in ABCG2, NQO-1, and HIF-1α expression, leading to PDT resistance in cells. In the TNYL-ICG-HAuNS group, ABCG2 expression could be maintained at almost normal levels. After repeated irradiation, TNYL-ICG-HAuNS still produced almost constant reactive oxygen species in the cells, while Nrf2 expression was significantly reduced (Figure 7B). Additionally, PDT resistance caused a significant decrease in the internalization of free ICG, but did not affect the cellular uptake of TNYL-ICG-HAuNS. The results suggest that TNYL-ICG-HAuNS can overcome photodynamic resistance in cancer cells and hold promise for simultaneous photothermal and photodynamic cancer therapy. Ye et al. prepared nanosponges by modifying platelet and neutrophil mixed cell membranes (PNM) on the surface of gold nanocages (AuNC) loaded with DOX and ICG in pores (Figure 7C). The nanosponges could capture and remove circulating tumor cells (CTCs) and tumor-derived exosomes through high-affinity membrane adhesion receptors, effectively disrupting the link between exosomes and immune cells. DOX and ICG were used for synergistic chemotherapy-photothermal treatment. In vitro, the nanosponges exhibited higher cellular uptake, deeper tumor penetration, and increased cytotoxicity against tumor cells compared to uncoated AuNCs or individually coated AuNCs [46]. Xie et al. reported the preparation of adjuvant monophosphatidyl lipid A (MPLA) lipid bilayer envelope and photosensitizer ICG loaded gold nanocages (MLI-AuNCs) for immunogenic PTT of aggressive melanoma (Figure 7D). The hollow porous AuNCs served as carriers for delivering MPLA and ICG while protecting ICG from photodegradation. Thermosensitive lipid bilayer encapsulated MLI-AuNCs exhibited uniform size, good biocompatibility and bioavailability, and prominent tumor accumulation, thereby improving the efficacy of PTT and PDT in gold nanocages loaded with ICG [47]. Yu et al. utilized the NIR- PTT effect of AuNCs and the temperature-sensitive phase change property of 1-tetradecanol (melting point of 39 °C) to develop a novel NIR-triggered co-release system of the chemotherapeutic drug DOX and the photosensitizer ICG for simultaneous chemotherapy, PTT and PDT of MDR human breast cancer MCF-7/ADR cells. The nanosystem, named DOX/ICG@biotin-PEG-AuNC-PCM, was constructed by filling the interior of AuNCs with DOX, ICG, and 1-tetradecanol and modifying the surface with biotinylated polyethylene glycol through Au-S bonding (Figure 7E). Under PBS at 40 °C or under 808 nm NIR irradiation (2.5 W/cm^2^), the co-release rate of DOX and ICG was significantly faster than at 37 °C (e.g., 67.27% or 80.31% vs. 5.57% of DOX, 76.08% vs. 3.83% of ICG for 20 min). The released ICG generated ROS for PDT [48]. Li et al. developed a light-responsive nanotherapeutic agent based on ICG conjugated mesoporous silica-coated gold nanobipyramidal (GNB@SiO_2_) for simultaneous fluorescence (FL) and photoacoustic (PA) imaging-guided PTT (Figure 7F). GNB@SiO_2_ with excellent photostability was used for PA imaging and PTT, while loaded ICG enabled FL imaging and PTT. Guided by FL/PA imaging, GNB@SiO_2_-ICG exhibited significantly enhanced therapeutic effects with complete clearance of tumor tissue and no tumor recurrence. This intelligent integrated dual-modality imaging and PTT-nanotherapy platform provides an efficient nanoplatform for cancer nanotherapy [49].

### 3.6. ICG/ Silica Nanocomposites

Core-shell nanoparticles composed of gold nanorod cores and silica shells have shown higher image contrast compared to “bare” gold nanorods in both photoacoustic imaging (PAI) and fluorescence imaging (FMI). These nanoparticles can be loaded with ICG to create bimodal imaging probes that overcome fluorophore quenching. The ICG-loaded gold nanorod–silica nanoparticles (Au@SiO_2_) exhibit low cytotoxicity and can maintain their photophysical properties in the near-infrared region. In one study by Kang et al., four types of nanoparticles containing encapsulated ICG were prepared and tested to enhance the PA signal. These nanoparticles included calcium silicate-encapsulated porous silicon nanoparticles (Ca-pSiNP-ICG composites), porous calcium silicate nanoparticles (CaS-ICG), microporous silica NPs sealed with calcium silicate (Ca-Silica-ICG), and liposomal NPs (Lip-ICG). It was found that the encapsulation of ICG in porous silicon, porous silica, or calcium silicate NPs significantly enhanced the PA response, with the porous silicon NPs (pSiNPs) showing the strongest enhancement [50]. These materials have low thermal conductivity and are biocompatible, making them suitable for in vivo applications. In another study by Dong et al., a bimodal photoacoustic fluorescence imaging nanoprobe was developed using ICG-loaded gold core-silica shell (Au@SiO_2_) nanoparticles [51]. The silica shell served as a spacer layer to prevent fluorophore quenching and maintain the photophysical properties of ICG. The nanoprobes were evaluated for in vivo imaging of tumor and ischemic mouse models using both PAI and FMI. The results demonstrated specific accumulation of the NPs in the tumor and ischemic regions, with detection achieved by both imaging modalities. Overall, these studies highlight the potential of core-shell NPs, particularly those incorporating silica shells, for enhancing the photoacoustic imaging contrast and maintaining the photophysical properties of ICG, enabling improved visualization of tissue physiology and pathology.

### 3.7. ICG-Based Multifunctional Composites

ICG is a biosafe photosensitizer capable of killing tumor cells by generating singlet oxygen and photothermal effects under near-infrared irradiation. However, it has limitations such as easy aggregation, rapid water degradation, and a short half-life. Combining ICG with NPs addresses these limitations effectively. ICG can be encapsulated not only with various organic nanomaterials (such as polymers, micelles, liposomes, dendrimers, and proteins) but also with inorganic nanomaterials (including magnetic, gold, mesoporous, calcium, and LDH-based materials) for drug delivery. Among these, colloidal mesoporous silica nanoparticles (MSNs) are one of the most mature and representative inorganic materials used in biology and medicine. They have transitioned from extensive in vitro studies to preliminary in vivo assays in small animal disease models. In contrast to other inorganic biomaterials like silica, carbon-based materials, and gold-based materials, calcium-based (CaXs) biomaterials can dissociate into non-toxic ions and participate in the normal metabolism of living organisms. Hence, this review focuses on the hybridization of CaXs biomaterials with inorganic/organic nanocarriers to achieve efficient ICG molecular delivery strategies.

**Figure 8:**
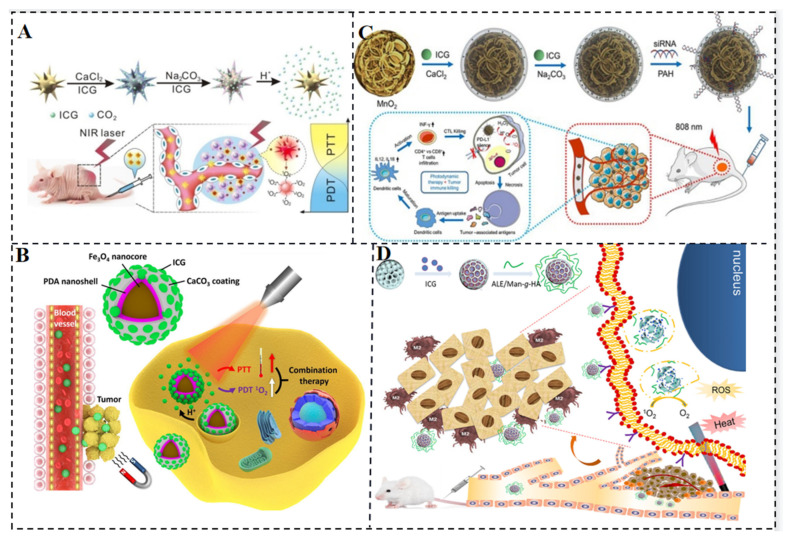
(**A**,**B**) Nanosystem consisting of a calcium carbonate carrier as a delivery ICG [52,53]. (**C**) Combination therapy system of MnO_2_-assisted ICG to enhance photodynamic effect and suppression of immune checkpoint PD-L1 by siRNA [54]. (**D**) Nanoplatform for apoptosis induced by thermal effects and cytotoxicity generated by ICG molecules and drug activity of tumor-associated macrophages [55].

CaXs biomaterials, including calcium phosphate, calcium carbonate, calcium silicate, and calcium fluoride, have found wide biomedical applications due to their excellent biocompatibility and biodegradability. Among them, calcium carbonate is employed for controlled drug release, leveraging its degradation in acidic environments to prevent drug inactivation and minimize side effects. Xue et al. reported GNS@CaCO_3_/ICG, a nanocomposite consisting of gold nanostars encapsulated by calcium carbonate containing ICG (Figure 8A). This composite combines the photothermal properties of gold nanostars (GNSs) with the photodynamic properties of ICG for combined PDT and PTT. Stable aggregates of ICG trapped by calcium carbonate on the GNS surface prevent ICG clearance and serve as pH reactors, enabling efficient local release of ICG drugs triggered by tumors. GNS@CaCO_3_/ICG exhibits enhanced antitumor effects under NIR irradiation in both in vitro and in vivo therapies, outperforming single PDT or PTT [52]. Moreover, GNS@CaCO_3_/ICG is utilized for tumor-targeted near-infrared fluorescence imaging, and its biodistribution and excretion pathways are monitored, demonstrating strong tumor specificity and tumor-triggered drug release capabilities.

Huang et al. employed a combined PTT-CT strategy using the near-infrared photosensitizer ICG, the photothermal conversion agent PDA, and the hypoxia-activated prodrug TPZ to address the issue of oxygen depletion during PDT. Under laser irradiation, ICG consumes oxygen and induces severe tumor hypoxia, activating TPZ drugs to damage DNA. Simultaneously, ICG generates reactive oxygen species that synergistically enhance the phototherapy efficiency with PDA. This strategy significantly improves cellular uptake and accumulation of ICG-PDA-TPZ NPs in tumors, exhibiting excellent antitumor properties against subcutaneous malignant glioma and in situ tumor models (Figure 8B) [53]. Nanocomposites such as Fe_3_O_4_@PDA@CaCO_3_/ICG (FPCI) NPs, which consist of calcium carbonate-modified magnetic polydopamine NPs loaded with ICG, have been developed to combine the photothermal ability of PDA with the photodynamic ability of ICG. Calcium carbonate not only encapsulates ICG in stable aggregates to evade blood clearance but also promotes controlled release in response to the acidic tumor microenvironment through self-decomposition. With the assistance of magnetic guidance, this multifunctional therapeutic agent enables the combination of PTT and PDT on tumors. To enhance PDT effects while inhibiting tumor cell resistance/escape, Liu et al. fabricated Mn@CaCO_3_/ICG nanoparticles that combine PDT and immunotherapy. These NPs were loaded with targeted PD-L1 siRNA (Figure 8C), resulting in improved PDT effects due to the protective effect of CaCO_3_ on loaded ICG and the oxygen generated by MnO_2_. The nanoplatform effectively delivers the loaded drug to tumor tissue, significantly improves tumor hypoxia, and exhibits a remarkable therapeutic effect by awakening the immune system through siRNA silencing of the checkpoint gene PD-L1 [54]. Gao et al. developed pH-sensitive mesoporous calcium silicate nanocomposites (MCNs) encapsulated with ICG for effective combination of PTT and PDT triggered by 808 nm NIR light (Figure 8D). ICG molecules generate cytotoxic ROS by transferring energy to surrounding oxygen under NIR light irradiation. These ROS, with their strong oxidative properties, destroy intracellular biomolecules and induce apoptosis. MCNs grafted with mannose and hyaluronic acid specifically target tumor-associated macrophages (TAMs) and tumor cells, promoting apoptosis both in vitro and in vivo [55].

In conclusion, the hybridization of calcium-based biomaterials with inorganic/organic nanocarriers provides effective strategies for ICG molecular delivery. This approach holds promise for tumor imaging, drug tracing, and antitumor therapy.

Metal-organic frameworks (MOFs) are hybrid porous polymers constructed from metal ions/clusters and multi-layered organic ligands. They possess unique properties such as a large surface area, tunable pore size and shape, tunable composition, and functionalized pore surfaces, which make them highly advantageous over conventional porous materials. These properties have led to their utility in applications such as drug adsorption, delivery, and release.

The advantages of polymeric materials such as biocompatibility, high stability and low inflammatory response can be combined with ICG to improve stability and retention in the organism.

Liposomal materials can improve biocompatibility while protecting ICG molecules from photodegradation.

Micelles can be employed as carriers to more effectively carry ICG molecules and other drugs into tissues and cells since they are small, simple to construct, and multifunctional.

Gold nanomaterials have superior photothermal properties as well as size homogeneity, in vivo metabolism can be regulated by material design, and they can also be made into hollow mesoporous structures to improve drug carrying capacity.

In conclusion, nanocomposites carry various drugs such as ICG to protect the drug from degradation while improving the biocompatibility, stability, and drug loading efficiency in the body to enhance the therapeutic effect.

The properties, functions, and therapeutic modalities of ICG nanocomposites are summarized as follows (Table 1):

## 4. Imaging and Light Therapy of ICG Molecules

### 4.1. Imaging and Light Therapy for Tumors

**Figure 9:**
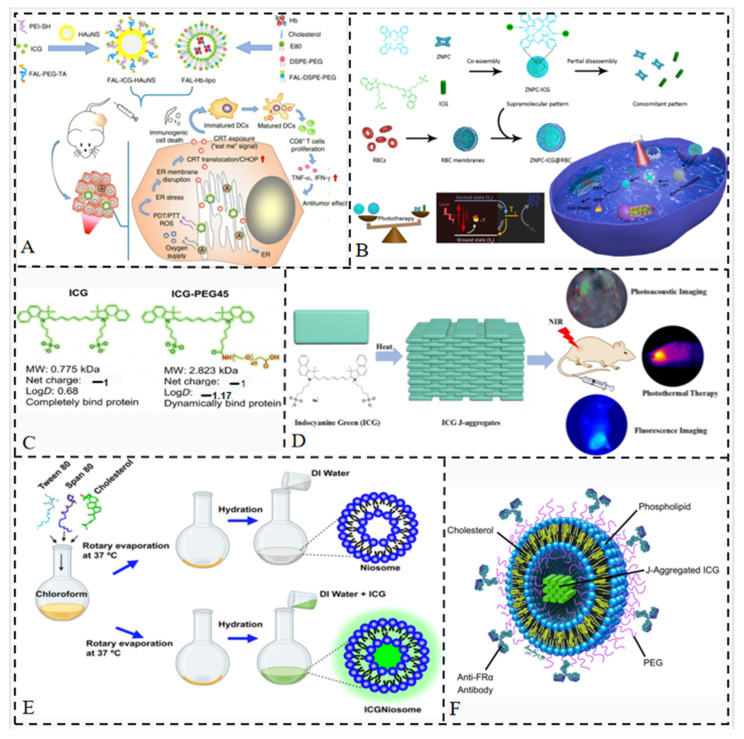
(**A**) A dual anti-tumor pathway nanosystem in which ROS damage the endoplasmic reticulum and induce a large release of calcium ions from the endoplasmic reticulum to stimulate other subcellular organelles such as mitochondria [31]. (**B**) Single phototherapy session in NIR by combining two photosensitizers [59]. (**C**) ICG-derived fluorophores excreted by the renal tubules [60]. (**D**) Schematic diagram of phototherapy after arrangement of ICG molecules into J-aggregates [63]. (**E**) Schematic diagram of the formation of biocompatible ICGNiosomes (ICGNios) that keep ICG molecules from being degraded in thicker tissues while maintaining strong fluorescence intensity [66]. (**F**) Schematic of the construction of a new PA contrast agent using ICG [67].

ICG is used in PTT and PDT due to its ability to absorb light energy and convert it into heat or generate singlet oxygen. However, there are challenges associated with using ICG in these therapies. One challenge is the exacerbation of tumor hypoxia caused by the tumor cell′s hypoxic microenvironment after PDT with ICG under light irradiation. This can hinder the effectiveness of tumor eradication. Another challenge is the limited immunogenicity of immunogenic cell death (ICD) induced by ROS generated through endoplasmic reticulum (ER) stress [56,57]. When ICG enters the cellular internalization, it mainly distributes in the cytoplasm, limiting its impact on ICD. To address these challenges, a dual ER-targeting strategy has been proposed by Li et al. [31]. The strategy combines PDT, PTT and immunotherapy using a nanosystem called FAL-ICGHAuNS (endoplasmic reticulum-targeted pardaxin-modified indocyanine green-fixed hollow gold nanospheres) and FAL-Hb lipo (oxygen-carrying hemoglobin liposomes) (Figure 9A). This strategy aims to reverse hypoxia in tumors. The FAL peptide-mediated hemoglobin targeting the ER replenishes oxygen under PDT hypoxic conditions and induces strong ER stress and calcium reticulum protein (CRT) exposure on the cell surface under NIR irradiation. This exposure triggers “eat me” signals that allow dendritic cells (DCs) to mature and present antigens to cytotoxic cells, leading to immunogenic cell death. The study showed that ER-targeted FAL-ICG-HAuNS exhibited higher cell-killing ability compared to non-targeted ICG-HAuNS.

To enhance ICG′s phototoxicity, it can be combined with another photosensitizer molecule. Zinc phthalocyanine (ZNPC), known for its admirable ROS production capacity, is considered a suitable candidate for combination with ICG [58]. Chen et al. developed a carrier-free nanoprobe called ZNPC-ICG using an ultrasound-assisted antisolvent precipitation method to co-assemble ZNPC and ICG. They further coated this nanoprobe with erythrocyte membrane to create a bionic carrier-free nanoprobe called ZNPC-ICG@RBC. This system enables desired PDT and PTT effects with improved physiological and optical stability of both ICG and ZNPC using a single NIR laser irradiation (Figure 9B) [59].

In clinical applications, ICG’s rapid clearance by the liver can limit its utility. To overcome this limitation, Du et al. developed ICG-PEG45, which is the first renal tubule-secreted NIR fluorophore. ICG-PEG45 selectively accumulates in renal cancer tissues with low expression of P-glycoprotein (P-gP) efflux transport protein [60,61], enabling highly specific fluorescence detection (Figure 9C) [60]. This approach shows promise for combining diagnosis and treatment and can potentially be used in conjunction with other modalities such as computed tomography (CT) [62].

Due to the rapid liver clearance of ICG molecules, their short retention time in tumors, and limited penetration depth, liposomal IJA has been investigated. In aqueous solution, ICG primarily exists as monomers and H-dimers, exhibiting absorption peaks at approximately 780 nm and 715 nm, respectively. Upon water bath heating at 65 °C, these peaks diminish, while a new absorption peak near 895 nm emerges, indicating the formation of J-aggregates. The J-aggregates are characterized by narrow absorption spectra (10–20 nm) and significant red shifts (~100 nm) due to the π-π stacking and electrostatic interaction arrangement of ICG molecules (Figure 9D) [63]. In vivo fluorescence imaging and ex vivo analysis of major organs and tumors demonstrated that within 48 h after injection, the J-aggregates were internalized by cells and converted back to ICG monomers, resulting in prolonged retention time at the tumor site and enhanced fluorescence intensity [64], thus improving the efficacy of imaging-guided cancer treatment. Another study revealed that the maximum fluorescence intensity was achieved at a lipid:ICG molar ratio of 250:1. The incorporation of ICG into liposomes not only enhanced fluorescence intensity, allowing detection up to 1.5 cm in muscle tissue, compared to only 0.5 cm with free ICG [65], but also facilitated deeper penetration into tumors and expanded detection limits. Moreover, liposomal encapsulation reduced fluorescence quenching caused by light and offered additional diagnostic value for early intervention in lymphatic abnormalities through near-infrared optical imaging. Bishnoi et al. developed ICGNiosomes (ICGNios) [66], a highly biocompatible, biodegradable, and easily ingested nanosized niosomal agent, to encapsulate and protect ICG from degradation. The preparation involved dissolving Tween 80, Span 80, and cholesterol in chloroform, followed by rotary evaporation at 37 °C to form a thin film (Figure 9E).

Thin films of nonionic surfactants were hydrated with deionized water, and ICG dissolved in deionized water was used to prepare surfactant vesicles and ICGNios. When tested in chicken breast tissue with a thickness of approximately 1 cm, ICGNios exhibited stronger fluorescence intensity compared to surfactant vesicles, which showed minimal emission at the same tissue thickness. Cayla A. Wood et al. introduced a novel method utilizing indocyanine green J-aggregate-based liposomes (PAtrace) as a photoacoustic contrast agent for targeting purposes (Figure 9F) [67]. They assessed the accuracy of spectral unmixing and targeting efficiency of PAtrace in a folate receptor α-positive ovarian cancer model in various settings, including mimetic, in vitro, and in vivo PA imaging. The results demonstrated that PAtrace outperformed monomeric ICG by significantly enhancing contrast quantification/sensitivity and enabling accurate estimation of SO_2_ levels. Given its performance characteristics and FDA-approved composition, PAtrace holds promise as a valuable reagent for future clinical molecular PA imaging.

### 4.2. Antibacterial Phototherapy

Antibiotics have been extensively utilized as the primary treatment method for bacterial infections. However, the emergence of antibiotic-resistant bacteria poses a challenge to traditional antibiotic therapy, making it difficult to completely eradicate bacteria and effectively manage bacterial infectious diseases. aPDT has emerged as a promising therapeutic approach, offering potential solutions to the issues of surgical invasiveness and antibiotic resistance [68,69,70]. By utilizing photosensitizers (PSs), aPDT can induce bacterial necrosis and apoptosis by generating large quantities of ROS upon exposure to light. These ROS can damage bacterial cell membranes and DNA [68,69,70]. Various PSs, including methylene blue, curcumin, dihydroporphyrin (Ce6), and porphyrins [71,72,73,74,75,76], can be activated by ultraviolet (UV) or visible light. However, there are two challenges that need to be addressed: the depth of infectious diseases within tissues and the limited tissue penetration ability of UV and visible light, which impose high requirements on the photosensitizers used in PTT.

NIR light offers several advantages, such as deeper tissue penetration, improved biosafety, and minimal background interference. As a result, NIR-responsive PSs have gained significant attention. ICG can generate abundant ROS when exposed to 808 nm NIR light, making it a promising candidate for NIR antimicrobial therapy. Despite the advancements in aPDT, two challenges hinder its clinical application. Firstly, aPDT relies on the presence of oxygen, but bacterial infections in deep tissue sites often exhibit an oxygen-deficient microenvironment, limiting the effectiveness of oxygen-dependent aPDT. Secondly, while aPDT can kill bacteria, it does not address bacterial-induced inflammation, which can lead to tissue damage. Therefore, there is a need for novel aPDT strategies that incorporate pro-oxygenation and anti-inflammatory properties.

**Figure 10:**
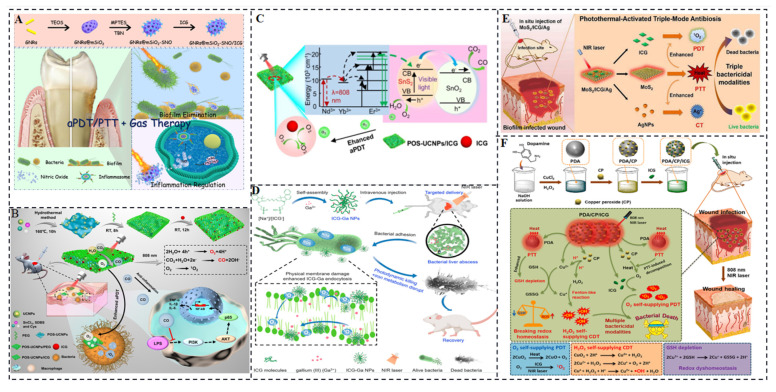
(**A**) Gold nanosystems for periodontitis and bacterial biofilms carrying ICG and nitrogen oxides [77]. (**B**,**C**) Upconversion nanosystem for dual gas therapy to modulate inflammation and enhance the photodynamic effect of ICG [78]. (**D**) Antimicrobial system of ICG-generated ROS disrupts bacterial membranes allowing gallium to penetrate the membrane causing disorders of iron metabolism [83]. (**E**) Therapeutic system for generating high-heat MoS_2_ nanosheets loaded with photothermal agents and antimicrobial Ag^+^ [84]. (**F**) Schematic of glutathione-depleting and hyperthermia-damaging biofilm [85].

Our group conducted a study on a nanoplatform, combining s-nitrosothiols (SNO) and ICG within mesoporous silica-coated gold nanorods (GNRs@mSiO_2_-SNO/ICG), to achieve NIR light-triggered aPDT with dual antimicrobial and anti-inflammatory functions for the treatment of periodontal disease. The nanoplatform exhibited PTT and gas therapy capabilities in addition to aPDT (Figure 10A) [77]. With the GNRs located internally, heat generated by the nanoplatform stimulated the release of nitric oxide (NO) before initiating PTT. The incorporation of the NIR-responsive photosensitizer ICG enables deeper tissue penetration and reduces damage to healthy tissues during aPDT for deep periodontal lesions. The generation of monoclinic oxygen by aPDT with ICG produces less harmful effects on healthy tissues. Moreover, the combination of NO gas molecules released by the NIR-responsive photothermal agent SNO and the ROS generated by ICG’s photodynamic action leads to the production of reactive peroxynitrite (ONOO-) molecules. These peroxynitrite molecules promote cell membrane peroxidation, enhancing the bactericidal effect of aPDT. In the aPDT/gas treatment group, there were fewer bacterial connections through bacterial fimbriae, cell wall rupture, leakage of contents, and a significant reduction in bacterial numbers compared to conventional mechanical debridement and antibiotic resistance combined with ICG treatment. This demonstrates that the NIR-responsive photosensitizer ICG can be applied to other challenging bacterial infectious diseases, particularly those affecting deep sites.

Despite the significant progress in aPDT, two challenges need to be addressed before its clinical application. Firstly, according to the mechanism of aPDT, photosensitizers can produce abundant singlet oxygen when oxygen is present and appropriate light is applied. However, bacterial infections at deep sites often have an oxygen-deficient microenvironment, limiting the effectiveness of oxygen-dependent aPDT. Secondly, aPDT solely targets bacteria and cannot eliminate bacterial-induced inflammation, which can cause damage to normal tissues. Therefore, novel aPDT strategies that address pro-oxygenation and anti-inflammation are greatly needed. In our group, Zhou et al. investigated a multifunctional aPDT nanoplatform (POS-UCNPs/ICG) mediated by near-infrared light by combining upconversion nanoparticles (UCNPs) with partially oxidized SnS_2_ (POS) nanosheets and ICG [78]. By utilizing a single 808 nm light source, aPDT can be achieved through ICG molecules, while POS nanosheets generate O_2_/CO through upconversion light excitation. During aPDT, the presence of O_2_ enhances the effectiveness of aPDT, while CO regulates inflammation via the PI3K/NF-κB pathway (Figure 10B,C). Consequently, the POS-UCNPs/ICG group exhibited the highest percentage of healing area in a mouse abscess model, reaching 91.55 ± 1.26%. In vitro and in vivo experiments provided evidence of the antibacterial activity and anti-inflammatory effect of POS-UCNPs/ICG. The therapeutic effect was further analyzed through immunofluorescence staining, Masson’s staining, hematoxylin-eosin staining, and colony-forming unit (CFU) assays. The results showed that the POS-UCNPs/ICG composites significantly accelerated recovery in an animal abscess model due to the synergistic effects of enhanced aPDT and anti-inflammatory treatment. This NIR light-responsive nanoplatform, which combines optimized antimicrobial capacity and immunomodulatory capabilities, holds great promise for clinical treatment of bacterial-induced infections.

In recent years, self-oxygenated aPDT nanoplatforms have gained attention for overcoming the limitations of low fluorescence yield and low singlet oxygen production under ICG light activation. These nanoplatforms utilize in vivo H_2_O_2_ catabolic oxygenation to enhance PDT [79,80,81,82]. Xie et al. developed ultrasmall non-antibiotic nanoparticles (ICG-Ga NPs) in a simple one-step process. These nanoparticles contain clinically approved Ga^3+^ and liver-targeted ICG molecules, aiming to eradicate multi-drug resistant (MDR) bacteria by harnessing the synergistic effects of PDT and iron metabolism blockade [83]. ICG can generate ROS upon near-infrared laser irradiation. The nanoscale coordinated particles based on ICG have demonstrated excellent performance in controlling bacterial infections, resulting in minimal antibiotic resistance. The ROS produced by ICG disrupts the membrane components of bacteria, allowing the antimicrobial gallium component to enter and displace iron in bacterial cells. This disruption of bacterial iron metabolism leads to synergistic bacterial killing and biofilm disruption (Figure 10D). ICG-Ga NPs demonstrated effective therapeutic efficacy against broad-spectrum β-lactamase *E. coli* (ESBL *E. coli*) and exhibited significant improvements in infectious liver abscess and keratitis treatment. Another study by Li et al. focused on the development of a photothermally activated antimicrobial nanoplatform called MoS_2_/ICG/Ag, which consisted of MoS_2_ nanosheets, ICG photosensitizers, and AgNPs. This nanoplatform offered triple CT/PTT/PDT for bacterial infections, including biofilm infections.

In the fabrication process depicted in Figure 10E, MoS_2_ nanosheets were obtained through exfoliation from bulk MoS_2_. Physical interaction, primarily electrostatic adsorption, facilitated the incorporation of ICG into the MoS_2_ nanosheets. Subsequently, AgNPs were covalently grafted onto the surface of MoS_2_ nanosheets via strong Ag-S bonds. The resulting MoS_2_/ICG/Ag nanocomposites demonstrated excellent stability in a physiological environment. Under 808 nm laser irradiation, the MoS_2_ nanosheets generated substantial heat, leading to the disruption of cell structures and the accelerated release of ICG and Ag^+^ (a commonly used chemical antibacterial reagent). The released ICG, aided by the 808 nm light, catalyzed the conversion of oxygen to singlet oxygen, thus achieving photodynamic sterilization. The presence of loaded ICG and AgNPs also enhanced heat generation, resulting in mutually reinforcing effects that produced combined or synergistic therapeutic effects. MoS_2_/ICG/Ag with its triple bactericidal mode exhibited broad-spectrum antimicrobial properties [84].

Significantly, MoS_2_/ICG/Ag exhibited remarkable inhibition of *S. aureus* biofilm formation and effectively eradicated bacteria residing deep within the biofilm. Furthermore, in vivo studies demonstrated the successful treatment of *S. aureus* biofilm-infected wounds using MoS_2_/ICG/Ag with minimal toxicity. Another study conducted by Xiao et al. focused on the rational integration of copper peroxide (CP) and indocyanine green into polydopamine NPs to create a multifunctional hybrid nanoplatform called PDA/CP/ICG [85]. This nanoplatform aimed to alleviate hypoxia within the microenvironment, thereby enhancing reactive oxygen species generation for efficient biofilm elimination.As illustrated in Figure 10F, when PDA/CP/ICG accumulated at the site of infection or within the biofilm, the acidic microenvironment triggered the simultaneous release of Cu^2+^ and H_2_O_2_. Cu^2+^ acted as a catalyst, converting the self-administered H_2_O_2_ into hydroxyl radicals (OH) through a Fenton-like reaction. This process also depleted the overexpressed antioxidant glutathione (GSH), further promoting oxidative stress. Additionally, the hyperthermia induced by PDA led to the decomposition of CP into O_2_ with the assistance of an 808 nm laser, which was immediately converted to ^1^O_2_ by ICG. Through the self-amplification of OH and ^1^O_2_ production, PDA/CP/ICG demonstrated satisfying antibacterial and anti-biofilm effects, along with excellent wound healing capabilities and favorable in vivo biosafety.

## 5. ICG for Angiography, Surgery, and Organ Reconstruction

**Figure 11:**
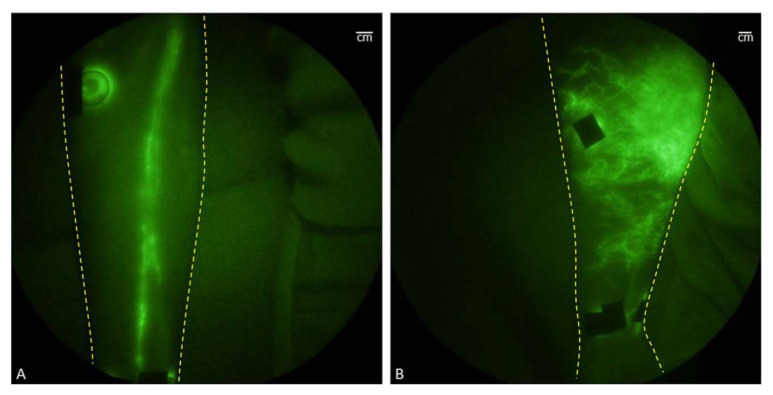
Near-infrared fluorescence imaging of healthy and affected limbs [86].

Under physiological conditions, upon intravenous injection, ICG molecules can undergo aggregation and polymerization when interacting with plasma proteins or lipoproteins. This process causes the main peak of the absorption spectrum to red-shift to around 805 nm or 810 nm, resulting in a relatively stable spectrum. It is worth noting that the fluorescence yield of ICG polymers is generally weaker compared to free or protein-bound ICG monomers. These properties make ICG molecules suitable for clinical applications as angiographic contrast agents, intraoperative visualization tools for tumor localization and resection, lymphatic imaging, and reducing the risk of false-positive resections. Near-infrared fluorescence (NIRF) imaging has shown promise in providing noninvasive in vivo imaging of human and animal lymphatic vessels [86]. NIRF imaging enables direct visualization of lymphatic vessels, and intracutaneous injection of ICG allows for the in vivo visualization of lymphatic transport, the NIR fluorescence imaging of healthy and affected limbs is shown in Figure 11A,B [86]. This technique can be utilized for early diagnosis of lymphedema, assessment of lymphatic function, and evaluating the response to lymphedema treatment (refer to Figure 11).

ICG is selectively taken up by hepatic parenchymal cells and excreted entirely into the bile, which is consistent with its lipophilic nature. This rapid elimination from circulation after intravenous injection is attributed to this characteristic. Therefore, ICG has also been studied for use in hepatectomy, particularly in addressing the technical challenges associated with liver resection in abdominal surgery and the specific challenges posed by cirrhotic liver resection due to impaired liver function and limited regenerative capacity [87].

To assess the risk of major hepatic resection, an ICG clearance test is performed. This test takes advantage of the fact that ICG dye is exclusively cleared by hepatocytes and excreted into the biliary system, providing information about liver mass and functional operational status. Additionally, the retention of ICG dye in the blood after injection can be used to stratify the risk of major hepatic resection. ICG lymphography has found applications in cancer detection, including breast, gastric, colorectal, anal, and skin cancers, among others [88,89,90,91,92,93]. It has shown high detection rates, low false-negative rates, and few cases of adverse reactions to ICG [94]. These various applications highlight the versatility and clinical potential of ICG in a range of medical procedures and diagnostic imaging techniques.

### 5.1. Angiography

Angiography using dyes such as fluorescein or ICG is valuable for evaluating retinal vascular diseases. While fluorescein angiography provides important information, it may not always capture certain aspects of the disease adequately. In such cases, digital indocyanine green video angiography (ICG-V) serves as a complementary technique to enhance fluorescein angiography and guide the treatment of choroidal neovascularization (CNV). ICG-V allows for a more detailed visualization of the retinal vasculature. When ICG is used in this technique, it can demonstrate hyperfluorescence or more commonly, hypofluorescence, in various ocular structures such as the cornea, vitreous, and pupil. This occurs due to the binding of ICG to the lipid component present in these structures. By enhancing the visualization of these areas, ICG-V assists in observing occult neovascularization, subretinal hemorrhage, or serous hemorrhagic fluid associated with neovascularization and neovascularization linked to pigment epithelial detachment. These poorly defined or occult vessels can be better imaged with the help of ICG-V. Occult CNV is a common finding in patients with exudative macular degeneration, particularly age-related macular degeneration. Since occult CNV cannot be clearly imaged using fluorescein angiography alone, researchers have utilized digital ICG-V as an additional technique in a study involving 657 consecutive eyes with occult CNV. The aim was to determine whether ICG-V could enhance the imaging of neovascularization and potentially increase the percentage of patients eligible for laser treatment [95].

ICG-V serves as an important adjunct to fluorescein angiography in the evaluation and treatment guidance of retinal vascular diseases, particularly for conditions where visualization of occult or poorly defined vessels is crucial. The use of ICG angiography as a complementary technique to fluorescein angiography has become increasingly common in ophthalmology. In cases where fluorescein angiography reveals advanced leakage associated with well-defined CNV known as type II occult CNV, ICG angiography can provide a more comprehensive depiction of the extent of the lesion and aid in the classification of CNV.

ICG angiography allows for the identification and characterization of various aspects of choroidal neovascularization, including focal points [29], plaques (whether well-defined or poorly defined), and combined lesions. Combined lesions can further be categorized into marginal spots (focal points at the edges of neovascularization-forming plaques), overlapping spots (hot spots covering neovascularization-forming plaques), or distant spots (focal points located away from neovascularization-forming plaques) [96,97]. By incorporating ICG angiography into the evaluation, clinicians can gain a more detailed understanding of the morphology and classification of choroidal neovascularization. This information is valuable for treatment planning and decision-making, particularly in cases where the lesion characteristics and extent are crucial factors.

Overall, the use of fluorescent molecule-based angiography, including ICG angiography, has become increasingly important and widely used in ophthalmology. It provides valuable insights into the vascular abnormalities and pathology of ocular diseases, aiding in diagnosis, classification, and treatment planning.

### 5.2. Surgery

Sentinel lymph node biopsy (SLNB) plays a crucial role in determining cancer spread and staging. Traditional tracers used for SLNB, such as blue dyes, nuclear proteins, nanocarbon, and radioactive technetium-99-labeled colloids or complexes, have limitations in terms of real-time monitoring and biosafety, potentially causing tissue damage.

ICG offers several advantages as a tracer for SLNB. It exhibits strong tissue penetration, high sensitivity, and non-invasiveness, with absorption and emission wavelengths around 765 nm and 820 nm, respectively. ICG is particularly suitable for real-time dynamic monitoring of SLN tracers and surgical navigation optical imaging due to its low molecular weight, non-toxicity, and rapid clearance through the liver without toxic accumulation in the body.

A study conducted by Chen et al. investigated the safety and efficacy of ICG NIR tracer-guided imaging during laparoscopic D2 lymph node dissection in patients with gastric cancer [98]. The study involved 266 subjects who were randomized into different groups. The results demonstrated that the mean number of retrieved lymph nodes in the ICG group was significantly higher compared to the non-ICG group. The ICG group also showed a higher number of perigastric and extragastric lymph nodes. Moreover, the mean total number of lymph nodes detected in the ICG group was significantly greater than the non-ICG group within D2 lymph node dissection.

Importantly, no significant complications or adverse effects were reported during postoperative recovery. These findings indicate that indocyanine green significantly improves the number of dissected lymph nodes and reduces lymph node noncompliance without increasing complications in patients undergoing D2 lymph node dissection. ICG fluorescence imaging can be incorporated as a routine technique for lymphatic mapping in laparoscopic gastrectomy, especially in total gastrectomy procedures. It enhances the detection rate and accuracy compared to conventional methods, potentially reducing the need for extensive lymph node dissection due to SLNB failure and lowering the risk of postoperative complications and recurrence. Overall, the use of ICG as a tracer in SLNB offers improved lymph node detection rates, accuracy, and patient outcomes.

In recent years, ICG NIR-FL imaging has emerged as an essential tool for real-time navigation during liver surgery. This technique has proven valuable in hepatobiliary surgery by aiding in the identification of subcapsular liver tumors, intrahepatic cholangiocarcinoma, and liver metastases. ICG accumulates in cancerous tissue of hepatocellular carcinoma and the non-cancerous liver parenchyma surrounding intrahepatic cholangiocarcinoma and liver metastases after preoperative intravenous injection, allowing for their visualization. Furthermore, ICG fluorescence imaging can provide enhanced visualization of extrahepatic bile duct anatomy and hepatic segmental boundaries, thus improving the accuracy of open and laparoscopic hepatectomy procedures. However, there are ongoing debates regarding the optimal use, dosage, and timing of ICG administration in clinical practice.

A systematic evaluation was conducted to address these controversies, involving the collection and analysis of 311 articles focused on open resections, laparoscopic, and robotic hepatectomy. The study specifically examined the dosing and time points of ICG administration, success rates of tumor detection and liver segmentation, as well as the impact of tumor/patient background and imaging settings [99]. The quality of the articles was assessed according to the Scottish Intercollegiate Guidelines Network (SIGN).

The results of the study indicate that, for tumor detection, the majority of studies utilized a dose of 0.5 mg/kg within 14 days before the surgery, with additional dosing at longer preoperative intervals (ranging from 0.02 mg/kg to 0.5 mg/kg). The reported tumor detection rate was 87.4%, while the successful liver segmentation fractionation rate was 88.0%. These findings suggest that the administration of ICG at a dose of 0.5 mg/kg within 14 days before surgery, with potential additional dosing at longer preoperative intervals, can achieve optimal liver staining results and improve tumor detection and liver dissection outcomes.

Overall, this systematic evaluation provides guidance on a relatively safe dose and timing of ICG administration in hepatobiliary surgery, facilitating improved visualization and enabling more accurate tumor detection and liver dissection. It helps to standardize the use of ICG in clinical practice for optimal surgical outcomes in liver surgery.

### 5.3. Organ Reconstruction

Intraurethral injection of ICG combined with NIR light visualization offers a real-time imaging method for visualizing the ureter [100]. This technique involves inserting the tip of a 6-F ureteral catheter into the ureteral orifice. Prior to undergoing robotic-assisted laparoscopic sacrovaginal fixation, a solution containing 25 milligrams of ICG dissolved in 10 mL of sterile water is injected through the open catheter. ICG binds reversibly to proteins on the ureteral cortex, leading to the staining of the ureteral lining. When excited by an NIR laser, the ICG molecules emit a green fluorescence, enabling the identification and localization of the ureter. One of the main advantages of this technique is that it only requires the insertion of the ureteral catheter’s tip [101]. Studies have demonstrated that the technique is safe, with results consistent with the safety profile of conventional intravenous and intra-biliary ICG injections. This method proves useful in various applications, such as identifying the edges of ureteral strictures during ureteral reconstruction. Additionally, it can help prevent medically induced ureteral injuries during pelvic surgery. By providing real-time visualization of the ureter, intraurethral ICG injection combined with NIR light imaging offers a valuable tool for enhancing surgical procedures involving the ureter and minimizing the risk of ureteral complications.

## 6. Outlook

ICG has advantages such as a good safety profile and absorption capacity in the NIR range, while it also has limitations, including its instability in aqueous solutions, rapid clearance in plasma, and low absorption in organisms. However, nanotechnology has emerged as a promising solution to address these challenges. Nanomaterials can be utilized to encapsulate ICG, providing several benefits. Firstly, they can protect ICG from decomposition and elimination, allowing for prolonged circulation time in the body. This is achieved through the enhanced EPR effect, which enables nanomaterials to passively accumulate in tumor sites or other disease sites. Nanomaterials can also enhance the chemical and photothermal stability of ICG, improving its performance. The use of ICG-containing nanoplatforms has shown great potential in the clinical treatment of tumors and bacterial infectious diseases. These nanoplatforms can be designed to specifically target the intended site, enhancing their effectiveness. Furthermore, by incorporating specific surface coatings and charges, the circulation time of ICG NPs can be tailored to suit specific applications. Biodegradable ICG NPs have emerged as an important alternative to overcome toxicity and accumulation issues associated with non-biodegradable materials.

In the field of bacterial infections, ICG-NP composites have demonstrated accelerated recovery in animal abscess models. This is achieved through a combination of enhanced aPDT and anti-inflammatory effects. The NIR photo-responsive nanoplatforms exhibit optimal antimicrobial capacity and immunomodulatory functions, making them promising for the clinical treatment of bacterial infections. Overall, the development of ICG-based nanomaterials holds significant potential in various applications, including diagnostics and therapy for tumors, bacterial infectious diseases, and other challenging conditions. Continued research and advancements in nanotechnology will contribute to the optimization and expansion of ICG nanoplatforms for clinical use.

## 7. Conclusions

ICG has the benefits of a good safety profile and near-infrared absorption, but it also has certain drawbacks, such as instability in aqueous solution, quick clearance in plasma, and limited uptake in living things. However, nanotechnology has become a potentially effective response to these problems. ICGs can be enclosed in nanomaterials, which has a number of benefits. They can extend ICG’s stay in circulation in the body and shield it from degradation and elimination. This is made possible by the improved EPR effect, which enables passive accumulation of nanomaterials at tumor or other disease locations. ICG’s chemical and photothermal stability is increased by nanomaterials, which also enhances the performance of ICG. Nanoplatforms that include ICG have a lot of promise for the clinical treatment of tumors and bacterial infectious illnesses. 

## Figures and Tables

**Table 1:** Summary of the properties, functions, and therapeutic modalities of ICG nanocomposites.

ICG Nanocomposites							
	Nanoplatforms	Functions	Types	Trigger	Target	Advantages	References
ICG/MOFs	ZSZIT	H2S gas therapy	vivo	PH		Large surface area/Adjustable pore size and shape/Adjustable composition/Functionalised surface	[14]
	DI@HMONs-PMOF	PDT/PTT	vivo	PH/660/808 nm			[15]
	DOX&ICG@H-PMOFm	CT/PTT/PDT&NIRF/PTI	vivo	PH/660&808 nm			[16]
	ICG-PtMOFs@GNSs@HSA-Gd	PT/FL/MSOT/X-ray CT/MRI	vivo	PH			[18]
	ZIF-ICG@ZIF-GOx@MPN	PTT/PDT/CDT	vivo	PH/NIR			[19]
	Fe-DOX@Gd-MOF-ICG	MRI/PTI/PAI&PTT/PDT	vivo	PH/NIR			[20]
ICG/Polymer	FA-ICG-PLGA-lipid	FLI	vitro	NIR		Biodegradable	[24]
	DOX&ICG-PLGA-lecithin-PEG	CT/ PTT	vitro	NIR			[26]
	ICG-PEG-PLL-PLLeu	FLI	vivo	NIR		Protection of enclosed drugs, high bioavailability, and good biocompatibility	[28]
Liposome-coated ICG	ICG -Lipid	PTT&Imaging	vivo	NIR			[29]
	FAL-ICG-HAuNS	PDT/PTT	vivo	NIR	ER		[31]
	Lipo-cyanine dyes	NIR-II Imaging	vitro	NIR-II			[32]
	FA-ICG&DOX-Gd-Lipo	PT/CT&MRI/FL/PAI	vivo	NIR	FRa		[33]
	ICG&Ce6&TPZ-Lipo	PTT/PDT	vivo	660/808 nm			[34]
	Lipo@ICG@CuS	PDT/PTT	vivo	NIR			[35]
ICG based Micelles Nanocomposites	Micelles -DOX & ICG	PTT/CT&NIR Imaging	vivo	PH/NIR/GSH	Nucleus/DNA Cleavage	Small size, Ease of assembly and versatility	[36]
	ICG-NH2-PEG-PCC	PTT&NIR Imaging	vivo	NIR			[37]
	GA-PEG-TK-ICG PMs	PTT/CT	vivo	NIR/ROS	HSP90		[38]
	ICG&PCL&5-FU&MEO2MA-b-HMAM	PTT/CT	vivo	NIR			[39]
	ICG-HA-PTX	PTT/CT	vivo	NIR	CD44		[40]
	TMTP1-PEG-PLGA-ICG	CT&NIR-FL	vivo	NIR	T-SLN		[41]
ICG/Gold Composites	TNYL-ICG-HAuNS	PDT/PTT	vivo	NIR	Nrf2&NQO-1&HIF-1α	photothermal effect, porous mesoporous structure, uniform size	[45]
	PNM@AuNC@ICG&DOX	PTT/Immunotherapy	vivo	NIR	Tumor-associated macrophage/neutrophil/Natural killer cell&CD44/VCAM-1/LFA-1		[46]
	MPLA & ICG-AUNCs	PTT/PDT	vivo	NIR	Tumor-associated antigen		[47]
	DOX/ICG@biotin-PEG-AuNC-PCM	PDT/CT	vivo	NIR	Endocytosis		[48]
	GNB@SiO_2_-ICG	PTT&FLI/PAI	vivo	NIR			[49]
ICG based multifunctional Composites	GN_S_@CaCO_3_/ICG	PT&FL Imaging	vivo	PH/NIR		Acid degradation, Immunomodulation	[52]
	Fe_3_O_4_@PDA@CaCO_3_/ICG	PDT/PTT	vivo	PH/NIR			[53]
	Mn@CaCO_3_/ICG-siRNA	PDT/Immunotherapy	vivo	PH/NIR	PD-L1		[54]
	ALE/Man-g-HA	PDT/PTT	vivo	PH/NIR	CD44		[55]

## Data Availability

Data supporting the findings are available from the corresponding authors upon reasonable request.
